# Cutting-Edge Machine Learning in Biomedical Image Analysis: Editorial for *Bioengineering* Special Issue: “Recent Advance of Machine Learning in Biomedical Image Analysis”

**DOI:** 10.3390/bioengineering11111106

**Published:** 2024-11-02

**Authors:** Sheng Lian, Zhiming Luo

**Affiliations:** 1 College of Computer and Data Science, Fuzhou University, Fuzhou 350108, China; 2 Department of Artificial Intelligence, Xiamen University, Xiamen 361005, China; zhiming.luo@xmu.edu.cn

Biomedical image analysis plays a critical role in the healthcare system. For doctors, it helps them to make more accurate evaluations and diagnoses of diseases, while for patients, it results in more sophisticated treatment plans and better rehabilitation opportunities. In recent years, the field of biomedical image analysis has undergone remarkable advancements, primarily fueled by the integration of cutting-edge machine learning (ML) techniques. Such an integration has revolutionized the way biomedical images are analyzed, leading to more accurate and efficient diagnostic capabilities. For instance, deep learning methods have become the mainstream technology in computer-aided diagnosis (CAD) schemes, aiming to help clinicians interpret medical images more efficiently and make diagnostic decisions in a more accurate manner [1,2].

This Special Issue (SI), titled “Recent Advance of Machine Learning in Biomedical Image Analysis (ML4BIA)” presents a carefully peer-reviewed and selected collection of research articles. These articles exemplify the advanced applications of ML in biomedical imaging, demonstrating innovative solutions to a spectrum of challenges currently tackled by the research community, including image quality assessment, segmentation, disease diagnosis and classification, and image processing for specific applications. These articles reflect the growing interest in ML-based biomedical image analysis. They not only showcase the technical aspects of ML in biomedical imaging but also provide insights into the real-world applications and their potential impact on healthcare. A brief summary is given below.

## 1. Disease Diagnosis and Classification in ML4BIA

In “A Synthesizing Semantic Characteristics Lung Nodules Classification Method Based on 3D Convolutional Neural Network”, Yanan Dong et al. integrate semantic characteristics, including lobulation, texture, diameter, etc., for the classification of lung nodules, boosting both diagnostic precision and model interpretability [3]. In “An Actinic Keratosis Auxiliary Diagnosis Method Based on an Enhanced MobileNet Model”, Shiyang Li et al. propose an enhanced MobileNet model that accurately differentiates actinic keratosis from non-actinic lesions, offering a promising tool for early skin cancer diagnosis [4]. Technically, it innovates by integrating customized preprocessing and network modifications, resulting in a highly efficient diagnostic method with potential for real-world clinical integration.

## 2. Image Quality Assessment in ML4BIA

In “Applying Self-Supervised Learning to Image Quality Assessment in Chest CT Imaging”, Eléonore Pouget and Véronique Dedieu innovatively harness self-supervised learning for chest CT image quality assessment, offering a significant advancement with the potential to optimize low-dose imaging protocols [5]. In “A Soft-Reference Breast Ultrasound Image Quality Assessment Method That Considers the Local Lesion Area”, Ziwen Wang et al. emphasize the importance of lesion regions in breast ultrasound quality assessment, adopting a novel soft-reference strategy for improved diagnostic insights [6].

## 3. Image Segmentation in ML4BIA

In “Semi-Supervised Medical Image Segmentation with Co-Distribution Alignment”, for the task of semi-supervised medical image segmentation (SS-MIS), Tao Wang et al. propose Co-DA, which addresses class imbalance and distribution mismatch by aligning class-specific distributions, and achieve promising results in SS-MIS tasks [7]. In “Radiomics-Based Quality Control System for Automatic Cardiac Segmentation: A Feasibility Study”, Qiming Liu et al. present a novel automated pipeline combining deep learning-based cardiac segmentation with radiomics-based quality control for both 2D and 3D cardiac images [8]. In “Semantic Segmentation of Gastric Polyps in Endoscopic Images Based on Convolutional Neural Networks and an Integrated Evaluation Approach”, Tao Yan et al. conduct exhaustive explorations using several CNN-based models for segmenting gastric polyps in endoscopic images, identifying UNet++ with a MobineNet v2 encoder as the best model, highlighting its clinical relevance for improved diagnosis [9].

## 4. Image Processing for Specific Applications in ML4BIA

In “High-Speed and Accurate Diagnosis of Gastrointestinal Disease: Learning on Endoscopy Images Using Lightweight Transformer with Local Feature Attention”, Shibin Wu et al. present FLATer, an efficient model that leverages local feature attention and vision transformers for superior endoscopic image classification [10]. In “Development of End-to-End Artificial Intelligence Models for Surgical Planning in Transforaminal Lumbar Interbody Fusion”, Anh Tuan Bui et al. introduce an AI-driven approach leveraging deep learning and machine learning to accurately predict surgical parameters from preoperative X-rays [11].

As this SI draws to a close, it is clear that the featured studies mark not only progress, but also a profound shift in the capabilities of medical diagnostics. The integration of machine learning has opened new avenues for innovation, with each contribution paving the way for more accurate, efficient, and personalized healthcare. Looking ahead, the potential for breakthroughs in this field is significant, promising an era where technology and medicine converge to enhance human life.

## References

[1] Chan H.P., Hadjiiski L.M., Samala R.K. (2020). Computer-aided diagnosis in the era of deep learning. Med. Phys..

[2] Zhang A., Xing L., Zou J., Wu J.C. (2022). Shifting machine learning for healthcare from development to deployment and from models to data. Nat. Biomed. Eng..

[3] Dong Y., Li X., Yang Y., Wang M., Gao B. (2023). A Synthesizing Semantic Characteristics Lung Nodules Classification Method Based on 3D Convolutional Neural Network. Bioengineering.

[4] Li S., Li C., Liu Q., Pei Y., Wang L., Shen Z. (2023). An actinic keratosis auxiliary diagnosis method based on an enhanced MobileNet model. Bioengineering.

[5] Pouget E., Dedieu V. (2024). Applying Self-Supervised Learning to Image Quality Assessment in Chest CT Imaging. Bioengineering.

[6] Wang Z., Song Y., Zhao B., Zhong Z., Yao L., Lv F., Hu Y. (2023). A soft-reference breast ultrasound image quality assessment method that considers the local lesion area. Bioengineering.

[7] Wang T., Huang Z., Wu J., Cai Y., Li Z. (2023). Semi-Supervised Medical Image Segmentation with Co-Distribution Alignment. Bioengineering.

[8] Liu Q., Lu Q., Chai Y., Tao Z., Wu Q., Jiang M., Pu J. (2023). Radiomics-Based Quality Control System for Automatic Cardiac Segmentation: A Feasibility Study. Bioengineering.

[9] Yan T., Qin Y.Y., Wong P.K., Ren H., Wong C.H., Yao L., Hu Y., Chan C.I., Gao S., Chan P.P. (2023). Semantic segmentation of gastric polyps in endoscopic images based on convolutional neural networks and an integrated evaluation approach. Bioengineering.

[10] Wu S., Zhang R., Yan J., Li C., Liu Q., Wang L., Wang H. (2023). High-Speed and Accurate Diagnosis of Gastrointestinal Disease: Learning on Endoscopy Images Using Lightweight Transformer with Local Feature Attention. Bioengineering.

[11] Bui A.T., Le H., Hoang T.T., Trinh G.M., Shao H.C., Tsai P.I., Chen K.J., Hsieh K.L.C., Huang E.W., Hsu C.C. (2024). Development of End-to-End Artificial Intelligence Models for Surgical Planning in Transforaminal Lumbar Interbody Fusion. Bioengineering.

